# Diagnostic value and correlation analysis of serum cytokine levels in patients with multiple system atrophy

**DOI:** 10.3389/fncel.2024.1459884

**Published:** 2024-09-04

**Authors:** Xueping Chen, Sihui Chen, Xiaohui Lai, Jiajia Fu, Jing Yang, Ruwei Ou, Lingyu Zhang, Qianqian Wei, Xiaoyan Guo, Huifang Shang

**Affiliations:** ^1^Department of Neurology, West China Hospital, Sichuan University, Chengdu, China; ^2^Department of Neurology, The Affiliated Hospital of Southwest Medical University, Luzhou, China

**Keywords:** multiple system atrophy, TNF-α, IL-6, diagnostic value, association analysis

## Abstract

**Background:**

The association between cytokines in peripheral blood and clinical symptoms of multiple system atrophy (MSA) has been explored in only a few studies with small sample size, and the results were obviously controversial. Otherwise, no studies have explored the diagnostic value of serum cytokines in MSA.

**Methods:**

Serum cytokines, including interleukin-6 (IL-6), interleukin-8 (IL-8), and tumor necrosis factor alpha (TNF-α), were measured in 125 MSA patients and 98 healthy controls (HCs). Correlations of these serum cytokines with clinical variables were analyzed in MSA patients. Diagnostic value of cytokines for MSA was plotted by receiver operating curves.

**Results:**

No significant differences were found in sex and age between the MSA group and the HCs. TNF-α in MSA patients were significantly higher than those in HCs (area under the curve (AUC) 0.768), while IL-6 and IL-8 were not. Only Hamilton Anxiety Scale (HAMA) has a positive correlation between with TNF-α in MSA patients with age and age at onset as covariates. Serum IL-6 was associated with HAMA, Hamilton Depression Scale (HAMD), the Unified MSA Rating Scale I (UMSARS I) scores, the UMSARS IV and the Instrumental Activity of Daily Living scores. However, IL-8 was not associated with all clinical variables in MSA patients. Regression analysis showed that HAMA and age at onset were significantly associated with TNF-α, and only HAMA was mild related with IL-6 levels in MSA patients.

**Conclusion:**

Serum TNF-α and IL-6 levels in MSA patients may be associated with anxiety symptom; however, only TNF-α was shown to be a useful tool in distinguishing between MSA and HCs.

## 1 Introduction

Multiple system atrophy (MSA) is a rare, rapidly progressing and fatal neurodegenerative disease, characterized by a variable combination of parkinsonism, cerebellar atrophy, and autonomic dysfunction of unknown etiology (Fanciulli and Wenning, 2015). Besides these core symptoms, many other non-motor manifestations also have been observed in MSA, including sleep dysfunction, constipation, depression, and anxiety (Zhang et al., 2018; Benrud-Larson et al., 2005; Lin et al., 2020). The clinical diagnosis requires the presence of autonomic dysfunction in combination with parkinsonian-like symptoms, poorly responsive to levodopa and/or cerebellar ataxia (Poewe et al., 2022). The neuropathological feature of MSA is the glial inclusion body composed of misfolded alpha-synaptic nucleoproteins (α-syn) (Fanciulli et al., 2019). The manifestation of MSA overlap with Parkinson’s disease (PD), other types of atypical parkinsonism, and other cerebellar disorders (Poewe et al., 2022; Gilman et al., 2000). In addition, MSA also presents with numerous non-motor symptoms, including sleep disturbances, constipation, depression, and anxiety. Extensive research indicates that mood disorders are common, particularly depression and anxiety in MSA patients (Zhang et al., 2018; Benrud-Larson et al., 2005; Ghorayeb et al., 2002). These symptoms can appear even before the onset of motor symptoms, posing challenges for early diagnosis of the disease (Zhang et al., 2018). Currently, there is still no effective treatment for MSA, and no objective markers for reference either. Therefore, identifying readily accessible and objective indicators to aid in the early diagnosis of MSA is significant. Furthermore, understanding the underlying mechanisms of these indicators can provide a theoretical foundation and research direction for the development of novel targeted therapies.

Although the etiology is unclear, many studies have shown that chronic neuroinflammation may be an important pathogenesis in MSA (Vieira et al., 2015; Jellinger, 2014; Ishizawa et al., 2008). Cytokines play a crucial role in neuroinflammation, particularly in the pathogenesis of MSA. Neuroinflammation typically accompanies neuronal damage and immune responses within the nervous system, leading to the release of inflammatory cells and immune molecules. Cytokines serve as signal transducers and regulators of immune responses in this process (Tang and Le, 2016). Dysregulation of neuroinflammation feedback in MSA would trigger microglial activation into effector phenotypes, namely M1 and M2. Once the M1 phenotype is activated, it will produce pro-inflammatory and cytotoxic molecules, including tumor necrosis factor alpha (TNF-α), interleukin-6 (IL-6), interleukin-1β (IL-1β), reactive oxygen species (ROS) and excitatory amino acids, which would induce more neuronal damage and cell dysfunction (Tang and Le, 2016). However, clinical studies focusing on inflammation in MSA are still relatively lacking. Starhof et al. found that cerebrospinal fluid (CSF) C-reactive protein (CRP), TNF-α, IL-6 and IL-1β levels in MSA patients were significantly higher than those in PD patients, and these cytokines have good diagnostic value in distinguishing MSA from PD (AUC 0.77, *p* = 0.007, 95% CI 0.660–0.867) (Starhof et al., 2018). However, this study did not investigate the association between inflammatory factors and clinical symptoms of MSA. A few studies examined the role of blood cytokines in MSA, but the sample sizes of these studies were relatively small and the results were conflicting (Jellinger, 2014; Kim et al., 2019; Brodacki et al., 2008; Kaufman et al., 2013).

Therefore, this cross-sectional study is aiming to: (1) examine the cytokine levels in the peripheral blood of MSA patients and health controls (HCs) with a large sample size, (2) verify the reliability of these serum cytokines as biomarkers in MSA diagnosis, (3) explore the correlations of these cytokines with clinical manifestations.

## 2 Materials and methods

### 2.1 Subjects

A total of 104 patients (58 male and 46 female) with MSA were recruited from the Department of Neurology in West China Hospital (WCH), the largest hospital in southwestern China, 21 MSA patients (10 male and 11 female) from the Affiliated Hospital of Southwest Medical University were also registered in our study, based on the diagnostic consensus for MSA published in 2008 (Gilman et al., 2008), and patients with “probable MSA” were included. Notably, our patients were followed up every 6 months, and diagnoses were confirmed at the follow-up visits. Specimens were collected when patients were identified as having “probable MSA.” Exclusion criteria were as follows: (1) PD, vascular parkinsonism, Progressive Supranuclear Palsy (PSP), Cortico-Basal Degeneration (CBD), and Dementia with Lewy Bodies (DLB); (2) having a family history of autosomal dominance; (3) acute or chronic inflammatory system or immune system diseases complicated with various potential infections; (4) patients taking non-steroidal anti-inflammatory drugs or glucocorticoids orally; (5) malignant tumors, or other life-threatening diseases; (6) recent history of trauma or head surgery; (7) education period less than 3 years.

We recruited 98 gender- and age-matched HCs from outpatient settings in WCH. HCs were primarily recruited from the family members of MSA patients to ensure alignment of their lifestyle factors. Clinical neurologists assessed the current health status of the HCs and the subject with a personal or family history of neurological degeneration was excluded.

The Research Ethics Review Committee of West China Hospital of Sichuan University approved the research protocol. All participants signed an informed written consent form for the use of their clinical data for scientific purposes after adequate explanation.

### 2.2 Clinical assessment

Trained researchers, verified for consistency, were responsible for conducting face-to-face interviews to collect detailed demographic information and assess relevant clinical presentations. In addition, the scale evaluators and the statistical data analysts were belonging to different group to avoid subjectivity. The Unified MSA Rating Scale (UMSARS) is a commonly used semi-quantitative rating scale to assess motor symptoms in patients with MSA (Krismer et al., 2022). The total score of UMSARS scale for all items in Part I and Part II were recorded. The Part III was autonomic nerve examination, which was achieved by neurogenic orthostatic hypotension (NOH) examination. We had the patients empty the bladder, lie flat for more than 10 min, and measured blood pressure in a quiet environment. Then, patients were instructed to stand immediately, and blood pressure was measured quickly at the 1st, 3rd, 5th, and 10th minutes after standing (Pavy-Le Traon et al., 2016). NOH was diagnosed if one or more of the following blood pressure changes were registered: systolic blood pressure (SBP) drop ≥20 mmHg from baseline, diastolic blood pressure (DBP) drop ≥10 mmHg from baseline, or SBP ≤90 mmHg regardless of how much blood pressure drops (Freeman et al., 2011; Brignole et al., 2018). The Part IV of the UMSARS quantitatively assesses the degree of incapacity on a scale of 0, 1, 2, 3, and 4 (0, patients completely able to take care of themselves; 1, patients need little help in daily life; 2, patients spend most time of the day in self-care and household tasks; 3, patients need a lot of help in daily life; 4, patients who are completely relying on caregivers). The Frontal Assessment Battery (FAB) and the Montreal Cognitive Assessment (MoCA) were used to examine the cognitive function (Royall, 2001; Nasreddine et al., 2005). The Hamilton Anxiety Scale (HAMD) and the Hamilton Depression Scale (HAMA) were used to assess patients’ emotional state (Ballesteros et al., 2007; Maier et al., 1988). The Epworth sleepiness scale (ESS) as a tool to assess daytime sleepiness, with a score of 10 indicating significant daytime sleepiness (Johns, 1991). The Parkinson’s Disease Sleep Scale (PDSS-2) and the Rapid Eye Movement Sleep Behavior Disorder Screening Questionnaire (RBDSQ) were used for sleep assessment including nocturnal disturbances and REM-sleep behaviors disorder (RBD) (Trenkwalder et al., 2011; Stiasny-Kolster et al., 2007). Fatigue was assessed in the MSA patients with Fatigue Severity Scale (FSS) (Friedman et al., 2010). The Non-Motor Symptom Scale (NMSS) was used to assess the overall aspect of a patient’s non-motor symptoms (Martinez-Martin et al., 2009). The Parkinson’s Quality of Living Questionnaire (PDQ39), Activity of Daily Living (ADL), Instrumental Activity of Daily Living (IADL) were used to assess quality of life of patients with MSA from different perspectives (Zhao et al., 2021; Pashmdarfard and Azad, 2020). We asked patients to rate their satisfaction for current life (0 for dissatisfaction, 100 for very satisfied) and recorded the scores as Subjective Life Satisfaction (SLS).

### 2.3 Measurements of peripheral blood cytokines

The peripheral blood samples from each MSA patient and HC were collected around 7:00 am, and all samples were sent to the laboratory center within two hours for testing. This study measured serum TNF-α, IL-6 and interleukin-8 (IL-8) levels, and enzyme-linked immunosorbent assay (ELISA) assay was used according to the kit-specific manufacturer’s instructions, and all data were recorded in pg./ml.

### 2.4 Statistical analysis

Demographic data were compared between MSA patients and HCs by applying Student’s *t* test for continuous variables and chi-square tests for categorical variables. IL-6, IL-8, and TNF-α levels were non-normally distributed in both the MSA group and the HCs in Kolmogorov–Smirnov one-sample test, even after the logarithmic transformation. Therefore, we used the Mann–Whitney U test to compare these cytokine levels in MSA patients and the HCs. Spearman correlation coefficient was used to evaluate the linear relationship between the clinical variables and cytokine levels. Spearman partial correlation analysis was used to adjust the potential influencing factors. Bonferroni correction was applied to optimize for multiple tests. A stepwise forward regression model was used, with IL-6 or TNF-α as dependent variable. Clinical variables, which had a *p*-value <0.15 in the correlation analysis, and other factors that were found to influence cytokine levels, were included as independent variables. The variance inflation factor (VIF) was used to assessed multicollinearity among independent variables.

A binary logistic regression model was used to further explore the association between cytokines and MSA. The presence or absence of MSA was set as the dependent variable, while age, TNF-α and IL-6 were set as covariates. The area under the curve (AUC) for TNF-α in MSA diagnosis were calculated with corresponding sensitivity, specificity, and 95% confidence intervals (CI). The data of the skewed distribution was described by the median and interquartile range (IQR), and the data with the normal distribution was described by mean and standard deviation (SD). SPSS 27.0 software and GraphPad Prism 9.0 were used in statistical analyses and statistical mapping, and a two-tailed signal difference at the *p* ≤ 0.05 level was applied.

## 3 Results

**Figure 1:**
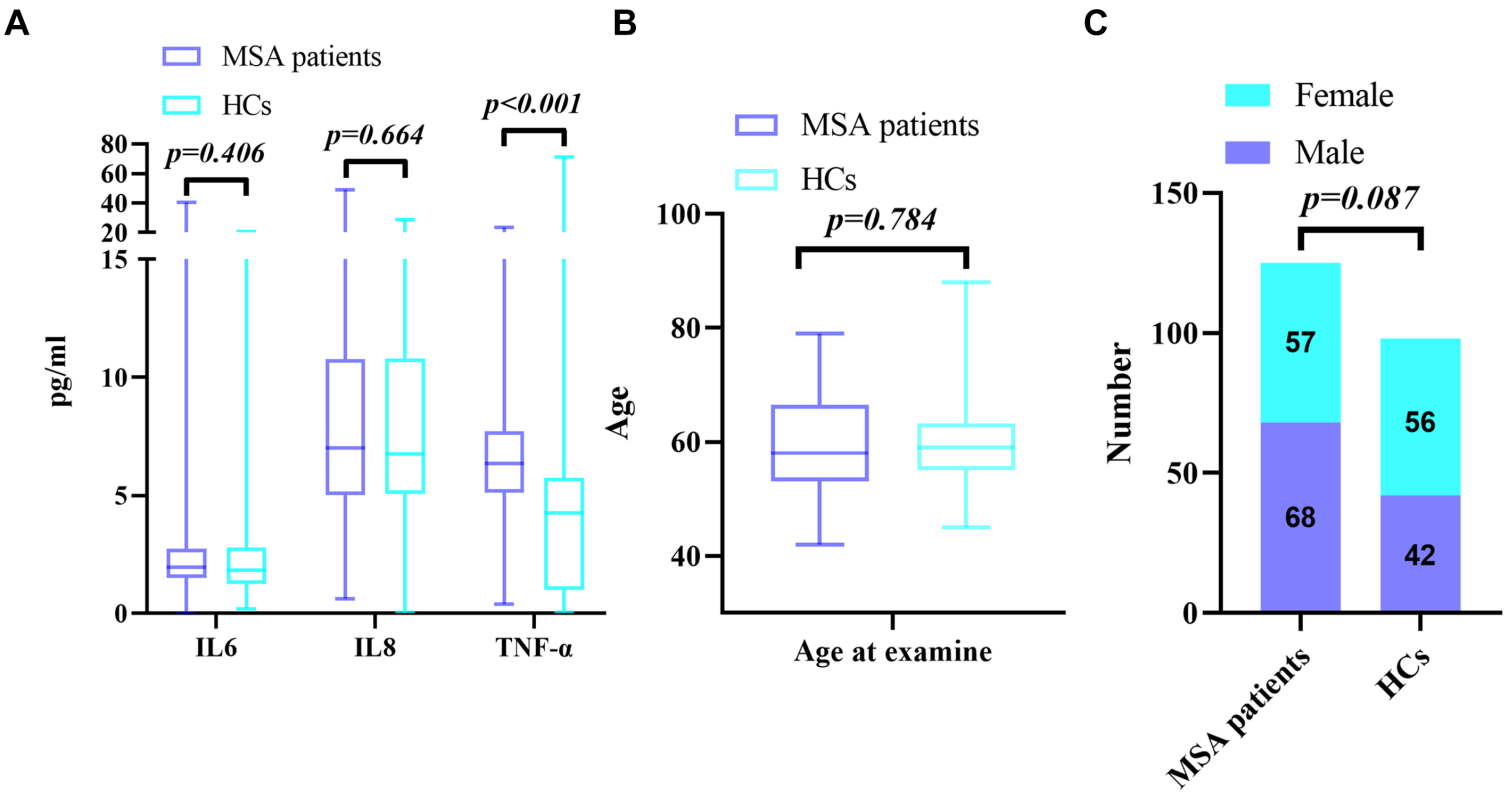
**(A)** Comparison of serum cytokine levels between MSA and HCs; **(B)** Comparison of age between MSA and HCs; **(C)** Comparison of gender distribution between MSA and HCs.

Clinical/demographic characteristics of MSA patients and HCs were shown in Table 1. It showed that there was no significant difference in the sex ratio (*χ*^2^ = 2.928, *p* = 0.087, Table 1 and Figure 1C) and age between MSA patients and the HCs (59.82 ± 8.42 vs. 59.54 ± 6.53, *p* = 0.784, Table 1 and Figure 1B). The Mann–Whitney U test showed that the serum TNF-α (median 6.36, IQR 5.13–7.72) levels in MSA patients were significantly higher than those in HCs (median 4.27, IQR 0.99–5.76, *p* < 0.001, Table 1 and Figure 1A). The levels of IL-6 (median 1.95, IQR 1.50–2.73) were higher in MSA patients than those in HCs (median 1.84, IQR 1.25–2.79, Table 1 and Figure 1A), but the difference was not statistically significant (*p* = 0.406). Serum IL-8 levels in MSA patients (median 7.00, IQR 5.03–10.78) were not statistically different from those in HCs (median 6.75, IQR 5.07–10.80, *p* = 0.664, Table 1 and Figure 1A). All these results were verified through Bonferroni multiple correction.

**Table 1:** Clinical/demographic characteristics of MSA patients and HCs.

Variables	MSA patients	Healthy controls	*p*-value
Age (years)	59.82 ± 8.42	59.54 ± 6.53	0.784
Sex (male/female)	68/57	42/56	0.087
age at onset (years)	58.08 ± 8.24	/	
Disease duration (years)	1.74 ± 1.31	/	
Educational level (years)	9.82 ± 4.12	/	
TNF-α	6.36 (5.13,7.72)	4.27 (0.99, 5.76)	<0.001
IL-6	1.95 (1.50, 2.73)	1.84 (1.25, 2.79)	0.406
IL-8	7.00 (5.03, 10.78)	6.75 (5.07, 10.80)	0.664
FAB	14.64 ± 4.02		
MoCA	21.21 ± 5.64		
Fatigue	29.80 ± 18.43		
PDSS-2	10.50 ± 7.73		
ESS	5.36 ± 4.62		
RBD	4.53 ± 3.60		
HAMD	10.26 ± 7.00		
HAMA	9.41 ± 6.17		
NMSS total	33.24 ± 28.45		
UMSARS I	12.30 ± 6.51		
UMSARS II	15.70 ± 6.80		
Orthostatic hypotension (yes/no)	47/78		
UMSARS IV(median, IQR)	2 (1, 2)		
PDQ-39	36.75 ± 27.48		
ADL	90.44 ± 16.19		
IADL	6.68 ± 1.92		
SLS	70.72 ± 18.60		

MSA, multiple system atrophy; FAB, frontal assessment battery; MoCA, Montreal cognitive assessment; PDSS-2, Parkinson’s disease sleep scale; ESS, Epworth sleepiness scale; RBD, REM-sleep behaviors disorder; HAMD, Hamilton depression scale; HAMA, Hamilton anxiety scale; NMSS, non-motor symptom scale; UMSARS, unified MSA rating scale; PDQ-39, Parkinson’s quality of living questionnaire; ADL, activity of daily living; IADL, instrumental activity of daily living; SLS, subjective life satisfaction; IQR, interquartile range.

Spearman correlation analysis showed no associations between serum IL-8 levels and clinical/demographic variables (Table 2). Several clinical/demographic variables in MSA patients were found to be correlated with serum IL-6 levels, including HAMA (*r* = 0.257, *p* = 0.004), HAMD (*r* = 0.220, *p* = 0.014), UMSARS I (*r* = 0.211, *p* = 0.018), UMSARS IV (*r* = 0.280, *p* = 0.002) and IADL scores (*r* = −0.230, *p* = 0.010), but not with other remaining variables. Serum TNF-α levels were correlated with HAMA scores (*r* = 0.217, *p* = 0.023), Fatigue (*r* = 0.176, *p* = 0.049), NOH (*r* = −0.194, *p* = 0.030), age (*r* = 0.259, *p* = 0.004), and age at onset (*r* = 0.270, *p* = 0.002), but were not related to other clinical variables, including MoCA, FAB, NMSS, UMSARS I, UMSARS II, UMSARS IV, HAMD, PDQ-39, PDSS-2, ESS, RBD, ADL, IADL, and SLS (Table 2). After adjusting for age and age at onset, Spearman partial analysis showed that serum TNF-α levels were only significantly associated with HAMA (*r* = 0.340, *p* < 0.001, Table 2).

**Table 2:** Clinical/demographic variables of MSA patients and the associations with inflammatory factor levels.

Variable	IL-8 (*p*-value)	IL-6 (*p*-value)	TNF-α (*p*-value)	TNF-α (after adjusting age and age at onset; *p*-value)
Age at onset (years)	0.953	0.079	**0.002**	
Age (years)	0.890	0.090	**0.004**	
Sex	0.228	0.886	0.855	0.661
Disease duration (years)	0.940	0.878	0.735	0.465
Educational level (years)	0.377	0.889	0.339	0.640
FAB	0.436	0.279	0.932	0.462
MoCA	0.222	0.278	0.551	0.840
Fatigue	0.095	0.063	**0.049**	0.124
PDSS-2	0.250	0.986	0.669	0.840
ESS	0.602	0.148	0.098	0.604
RBD	0.283	0.748	0.563	0.997
HAMD	0.637	**0.014**	0.100	0.098
HAMA	0.475	**0.002**	**<0.001**	**<0.001**
NMSS total	0.222	0.199	0.123	0.165
UMSARS I	0.355	**0.018**	0.601	0.392
UMSARS II	0.502	0.138	0.836	0.762
UMSARS IV(stage)	0.117	**0.002**	0.310	0.566
NOH (yes/no)	0.306	0.863	**0.030**	0.566
PDQ-39	0.539	0.060	0.288	0.223
ADL	0.529	0.050	0.575	0.613
IADL	0.686	**0.010**	0.784	0.262
SLS	0.232	0.144	0.855	0.944

MSA, multiple system atrophy; FAB, Frontal assessment battery; MoCA, Montreal cognitive assessment; PDSS-2, Parkinson’s disease sleep scale; ESS, Epworth sleepiness scale; RBD, REM-sleep behaviors disorder; HAMD, Hamilton depression scale; HAMA, Hamilton anxiety scale; NMSS, non-motor symptom scale; UMSARS, unified MSA rating scale; PDQ-39, Parkinson’s quality of living questionnaire; NOH, neurogenic orthostatic hypotension; ADL, activity of daily living; IADL, instrumental activity of daily living; SLS, subjective life satisfaction. Spearman correlation analysis showed no associations between serum IL-8 levels and clinical/demographic variables; HAMA, HAMD, UMSARS I, and UMSARS IV scores were found to be related to IL-6 levels; several clinical/demographic variables in MSA patients were found to be correlated with serum TNF-α levels, including age and age at onset. After adjusting age and age at onset, spearman partial analysis only showed that HAMA was significantly associated with TNF-α levels. Bold values mean that the differences are statistically significant.

In the stepwise forward regression analysis, the age, age at onset, UMSARS I, UMSARS II, UMSARS IV, PDQ-39, Fatigue, ESS, HAMD, HAMA, ADL, IADL and SLS were used as independent variables, and IL-6 was set as dependent variables; the results showed that only HAMA was related to serum IL-6 levels ([*F* = 8.687, *p* = 0.004, *R*^2^ = 0.066, Adjusted *R*^2^ = 0.058]). With age, age at onset, Fatigue, HAMA, HAMD, ESS, NMSS and NOH as independent variables, we found that TNF-α was associated with HAMA and age at onset (*F* = 13.535, *p* < 0.001, *R*^2^ = 0.182, Adjusted *R*^2^ = 0.168). There was no multicollinearity among all included independent variables (all VIF < 1.0).

**Figure 2:**
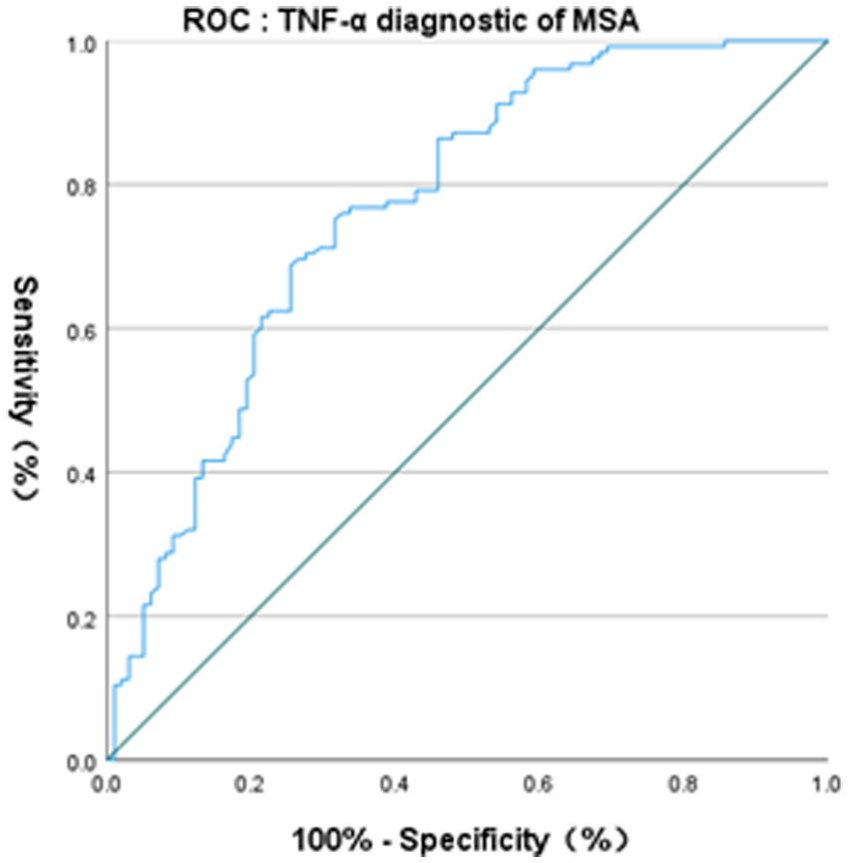
ROC curve of serum TNF-α for distinguishing HCs and MSA.

The binary logistic regression model showed that only TNF-α was positively associated with the presence or absence of MSA (OR 1.237, *p* < 0.001). The Receiver operating characteristic curve (ROC) curve of TNF-α for the diagnosis of MSA showed AUC was 0.768 (*p* < 0.01, 95% CI 0.705–0.832). The critical value of TNF-α for the diagnosis of MSA was 5.125 pg./mL, with a diagnostic sensitivity of 75.2% and a specificity of 68.4% (Figure 2).

## 4 Discussion

In the present study, we found a pronounced increase in the serum levels of TNF-α in MSA patients compared with HCs. Furthermore, the TNF-α serum levels in MSA patients were found to be associated with HAMA. In addition, ROC curve analysis indicated that TNF-α could discriminate MSA patients from HCs, as a screening diagnosis biomarker. To the best of our knowledge, this is the first study to investigate the diagnostic value of serum cytokines in MSA patients, with a large sample size.

### 4.1 Serum levels of TNF-α was elevated in MSA patients

TNF-α, as a main pro-inflammatory cytokine, is one of the key regulators of the inflammatory response and is mainly synthesized by activated microglia in the central nervous system (CNS) during neuroinflammatory (Lieberman et al., 1989). Studies have found that TNF-α released by microglia plays a major role in angiotensin induced dopaminergic cell and oligodendrocyte death through multiple mechanisms (Probst-Cousin et al., 1998; Borrajo et al., 2014; Ndayisaba et al., 2019). A previous genetic study showed that TNF-α may have a toxic role in the pathophysiological process of MSA (Nishimura et al., 2005). Lenalidomide can reduce the proliferation of astrocyte, microglia and the level of α-syn by inhibiting the production of TNF-α in MSA mice, and the effect of lenalidomide can partially improve the motor dysfunction of MSA mice (Valera et al., 2017). These findings support TNF-α as an important biomarker in MSA. Our results showed that MSA patients had higher TNF-α than HCs, which is consistent with the results of two previous studies (Brodacki et al., 2008; Kaufman et al., 2013). One previous study also found that the higher TNF-α levels were associated with mild motor symptoms and early stage of disease (Kaufman et al., 2013). However, another study found no association between TNF-α and MSA, probably due to the long disease duration (3.4 ± 1.7 years) in the recruited MSA patients (Kim et al., 2019). Studies have shown that neuroinflammation could be an early event in the pathogenic mechanism of MSA, and in the early stage of neurodegeneration, increased cytokine levels may reflect a compensatory physiological response to neuronal injury with potential neuroprotective purposes, whereas in the middle or late stage of the disease, this protective mechanism may be markedly attenuated (Müller et al., 1998; Ahmed et al., 2012; Stefanova et al., 2007; Yamasaki et al., 2017). In the present study, the disease duration was 1.74 ± 1.31 years, indicating that our patients were mostly in the early stage of MSA. We found that the TNF-α levels were significantly higher than those in HCs, and this result can be partially explained by the relatively short disease duration in our MSA patients. Furthermore, in studies using CSF samples, TNF-α were also found to be significantly increased in MSA patients compared to PD or normal controls, and CSF TNF-α was even considered an important cytokine in MSA (Starhof et al., 2018; Compta et al., 2019; Fellner and Wenning, 2019). Although this study used serum samples, it was found that serum TNF-α can directly cause blood–brain barrier dysfunction by reducing extracellular resistance and cell polarity to induce or aggravate neurodegenerative diseases (Sweeney et al., 2019; Yang et al., 2022; O’Carroll et al., 2015; Murdaca et al., 2022). Therefore, our study reported that elevated TNF-α levels in serum may have potential as a biomarker in MSA, especially in the early stage of disease.

In this study, we did not find any significant differences in IL-6 levels between MSA patients and HCs, and this finding was not consistent with other studies which found higher IL-6 levels in MSA (Brodacki et al., 2008; Kaufman et al., 2013; Hall et al., 2018). However, these previous studies also had found that IL-6 levels were also increased in PD patients compared to HCs, while TNF-α levels were not increased, so the author emphasized that the observed increase of IL-6 levels may not be specific for MSA (Kaufman et al., 2013; Hall et al., 2018). Different from the above-mentioned studies, we collected serum samples after an overnight fast to avoid the influence of some pro-/anti-inflammatory diets on circulating cytokines (Emerson et al., 2017; Sun et al., 2022). In addition, patients treated with NSAIDs were included in one previous study, but we excluded this interference in our study (Brodacki et al., 2008). Notably, that the sample size in these two studies were relatively small, with no more than 20 cases. We did not find any differences in serum IL-8 levels between MSA patients and HCs. Till now, there were no studies on IL-8 levels in serum of MSA patients. In CSF, one study found that the CSF IL-8 levels in MSA patients were higher than those in the HCs, but another study reported that the CSF IL-8 levels in MSA patients were significantly lower than those in the control group (Yamasaki et al., 2017; Hall et al., 2018). These findings suggested that CSF IL-8 levels may fluctuate dramatically in MSA patients. In addition, the different inclusion and exclusion criteria for these studies may partially explain the inconsistence. In summary, our findings indicated that serum IL-6 and IL-8 may have low value in diagnosis of MSA.

### 4.2 Diagnostic value of serum TNF-α for MSA

To our knowledge, this is the first study to investigate the diagnostic value of serum cytokines for MSA. As confirmed by ROC curves, TNF-α levels exhibited high values of AUC, and the sensitivities and specificities of TNF-α were also significant, indicating that TNF-α may be a reliable biomarker for the diagnosis of MSA. MSA patients typically exhibit signs of immune system activation and neuroinflammation. Elevated levels of TNF-α may reflect this inflammatory state (Stefanova et al., 2007). Moreover, TNF-α not only plays a crucial role in inflammatory responses but may also impact neuroprotective mechanisms and neuronal function, thereby also relating to the clinical symptoms of MSA (Stefanova and Wenning, 2016). Thus, TNF-α is involved in the pathogenesis of MSA. It is worth noting that studies have shown that CSF levels of TNF-α in patients with MSA were significantly higher than those in PD or HCs, regardless of the duration and subtype (MSA-P or MSA-C) (Starhof et al., 2018; Compta et al., 2019). CRP combined with a cytokine set (including TNF-α) in CSF was also found to have the best diagnostic value for discriminating PD (AUC = 0.77, *p* = 0.007, 95% CI 0.660–0.867) (Starhof et al., 2018). Therefore, in the future studies, combination of serum TNF-α with other inflammation factors may have higher diagnostic value in MSA.

### 4.3 Serum levels of TNF-α and Il-6 were associated with the HAMA scores in MSA patients

Anxiety, as one of the most common emotional disorders in MSA patients, usually leads to multiple hospital admissions because of excessive worry about their disease, which increases the disease burden, occupies the medical resources, and affects the quality of life of patients (Schrag et al., 2010). Our previous studies found that anxiety in MSA patients was related to uncontrollable factors, including gender (female), longer disease duration and disease severity (Zhang et al., 2018). The present study found that elevated TNF-α levels was a risk factor for anxiety mood in MSA patients even after adjusting age and age at onset (*r* = 0.340, *p* < 0.001). Stepwise forward regression analysis also confirmed a positive association of TNF-α with HAMA score and age at onset. These findings illustrate the possible involvement of TNF-α in the progression of anxiety symptoms in MSA patients. A previous study did not found any significant correlations between cytokines and non-motor symptoms in MSA patients, but this study only included 14 MSA patients (Kaufman et al., 2013). In addition, anxiety has been found to be significantly positively correlated with TNF-α levels in related studies focusing on PD patients (Wang et al., 2016; Lindqvist et al., 2012; Menza et al., 2010). Furthermore, severe anxiety and depression can also induce immune activation, leading to elevated levels of cytokines, including TNF-α, and the use of immunosuppressants such as interferon-α can alleviate these emotional disorders (Anisman, 2009). Another study included 70 patients with generalized anxiety disorder (GAD), and found that GAD patients had significantly elevated serum cytokines, including TNF-α, but serum TNF-α levels were significantly reduced after successful treatment of anxiety disorders (Hoge et al., 2018). These findings suggest that chronic inflammation may affect the anxiety mood in MSA patients. Our study also found a positive correlation between serum TNF-α levels and age at onset in MSA patients, which is also consistent with previous findings that serum TNF-α levels were related to aging and significantly higher in older adults than in younger adults (Nozu et al., 2018; Chen et al., 2023; Wyczalkowska-Tomasik et al., 2016).

Although correlation analysis found that IL-6 was linearly associated with multiple clinical variables, stepwise forward regression analysis found only HAMA had positive association with IL-6 level, which suggested that IL-6 had some effect on anxiety in MSA patients. Previous studies also have found that IL-6 was also associated with many non-motor symptoms in PD patients including anxiety and depression (Selikhova et al., 2002; Veselý et al., 2018). Some studies have found that serum IL-6 level may be related to anxiety symptoms in other diseases, and the use of IL-6 antagonist can significantly improve these anxiety symptoms (Tiosano et al., 2020; Zou et al., 2020; Yang et al., 2017). These studies also seem to illustrate that IL-6 may be involved in the pathogenesis of anxiety symptoms. From a mechanistic perspective, TNF-α and IL-6 are primary inflammatory mediators in the immune system (Connolly et al., 2021). Elevated levels of TNF-α and IL-6 may inhibit the synthesis and release of neurotransmitters such as serotonin and dopamine, thereby leading to mood dysregulation disorders like anxiety. Inflammatory responses can alter the expression and signaling of neurotransmitter receptors such as NMDA receptors and γ-aminobutyric acid (GABA) receptors, influencing mood regulation and cognitive function. TNF-α and IL-6 may contribute to the onset of anxiety symptoms in MSA patients through these mechanisms (Khandaker et al., 2015; Pariante, 2017). Therefore, suitable clinical intervention on the anxiety may have potential benefits in MSA by monitoring the serum TNF-α and IL-6 levels.

## 5 Limitations

There are several limitations in this study. (1) Due to the cross-sectional design of this study, we were unable to further reveal the causal relationship between TNF-α and IL-6 levels and anxiety symptoms in MSA patients. (2) All diagnoses were based on clinical diagnosis. Although we ensured the diagnosis of MSA by following the patients for at least six months, the possibility of misdiagnosis still existed due to the lack of neuropathological basis. (3) The participants were only recruited from two tertiary hospitals in China, and further results of a multicenter study will be needed in the future.

## 6 Conclusion

In summary, this study found that serum TNF-α levels was increased in MSA patients compared to those in HCs. Serum TNF-α may serve as a biomarker in diagnosis of MSA. In addition, the elevated TNF-α and IL-6 levels were associated with anxiety symptoms in MSA patients, and further studies with longitudinal and prospective designs from multiple centers and ethnic populations are required to verify these preliminary findings.

## Data Availability

The raw data supporting the conclusions of this article will be made available by the authors, without undue reservation.
