# Supplementary figures and images for: Diagnostic value and correlation analysis of serum cytokine levels in patients with multiple system atrophy
Source: Front Cell Neurosci. 2024 Sep 4;18:1459884. doi: 10.3389/fncel.2024.1459884 (PMC11409425; doi:10.3389/fncel.2024.1459884)

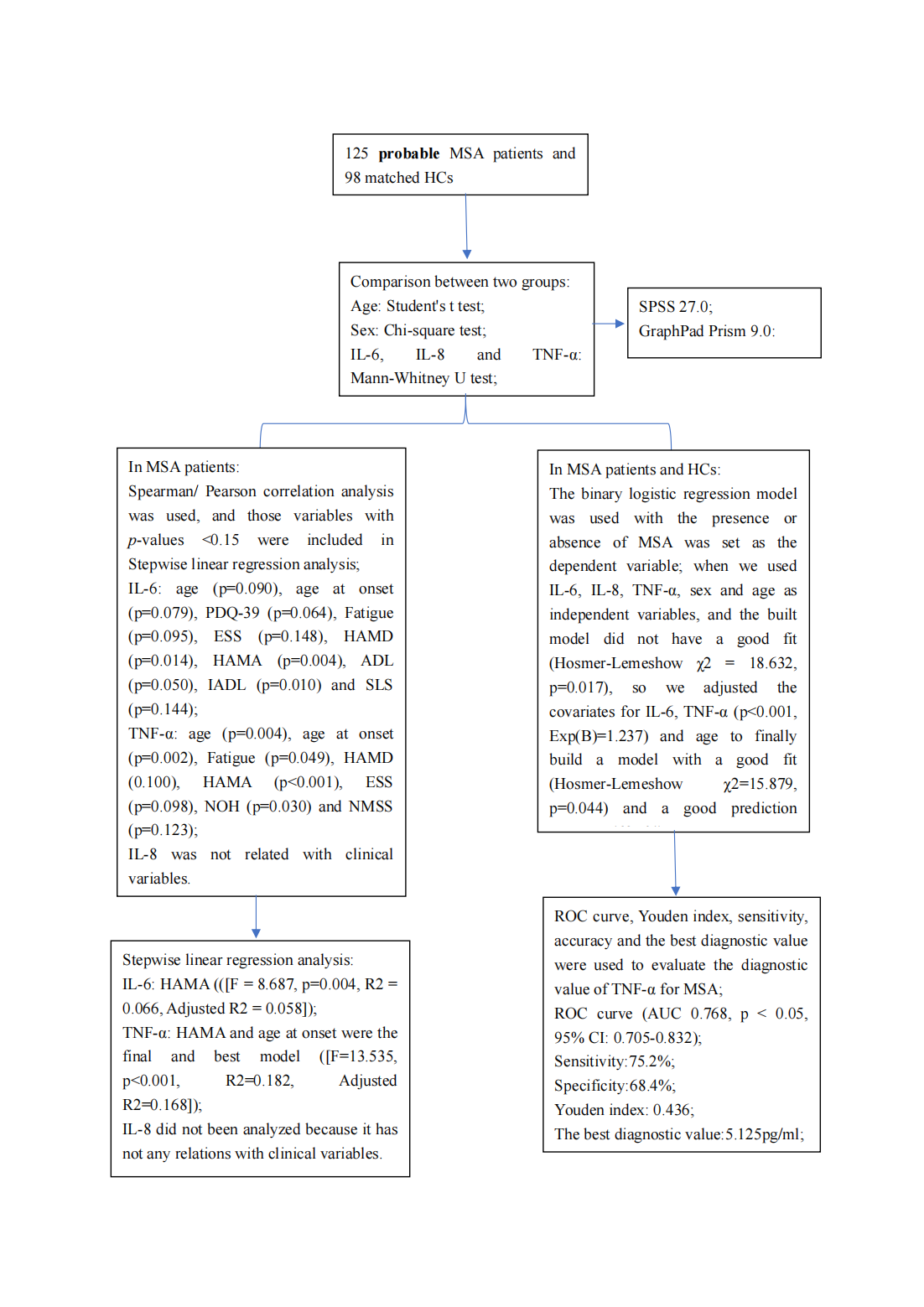

Supplement: Supplementary file 1 [file Image_1.TIF]
